# Training Peer Support Workers in Mental Health Care: A Mixed Methods Study in Central Catalonia

**DOI:** 10.3389/fpsyt.2022.791724

**Published:** 2022-04-08

**Authors:** Gemma Prat Vigué, Ivan Cano Prieto, Ruben del Río Sáez, Rut Vilanova Masana, Salvador Simó Algado

**Affiliations:** ^1^Althaia Xarxa Assistencial Universitària de Manresa, Manresa, Spain; ^2^Fundació Osonament, Vic, Spain; ^3^Universitat de Vic - Universitat Central de Catalunya, Barcelona, Spain

**Keywords:** peer support (PS), mental health, personal recovery, social innovation, mixed method approach

## Abstract

**Introduction:**

A mental health peer support program was implemented at two reference institutions in Central Catalonia. The program culturally and contextually adapted successful international projects by training people with experience of mental health problems and ensuring their employment in multidisciplinary health care teams. This study explores the influence of peer interventions in mental health on the three groups of participants: peer support workers, service users, and mental health professionals.

**Methods:**

A mixed observational method design included pre-, inter-, and post-experimental components and a qualitative description of the impact. The triangulation of the qualitative and quantitative findings showed its coherence and facilitated the understanding of the results. Outcomes and measures were as follows: self-stigma (Self-Stigma Questionnaire); life satisfaction (Scale of Satisfaction with Life); participation in relevant activities (Engagement in Meaningful Activities Survey); personal recovery (Scale-revised Recovery Assessment); occupational performance (Canadian Occupational Performance Measure); and attitudes toward mental illness (Community Attitudes toward Mental Illness).

**Results:**

The program showed beneficial effects on peer support workers' (PSW) perceptions of occupational performance, specifically on the ability to find work (*p* = 0.038), work as a peer support worker (*p* = 0.016), give to the community (*p* = 0.011), and satisfaction in the ability to find work (*p* = 0.031). The assessment made by the three groups of participants was very positive: the PSWs showed an increase in self-esteem and a feeling of usefulness; users of the service described the experience as a source of hope and optimism in their recovery process; and professionals described the program as a positive step in their professional growth.

**Discussion:**

The peer-to-peer strategy is a source of hope in the personal recovery process, providing meaning to life for the PSWs while providing an extra source of support to service users in their process of personal recovery. The results offer us lines of improvement for future implementations. PSW's final emphasis has us reflecting on improvements to enhance their own wellness in mental health care services. The findings show the importance of working on life projects and their impact on the recovery process.

## Introduction

One of the principles of community-based mental health care is personal recovery orientation (1). According to Anthony (2), “recovery is described as a deeply personal, unique process of changing one's attitudes, values, feelings, goals, skills, and/or roles. It is a way of living satisfying, hopeful, and contributing life even with limitations caused by illness. Recovery involves the development of a new meaning and purpose in one's life as one grows beyond the catastrophic effects of mental illness”. Until now, recovery has been understood exclusively as the resolution of the clinical aspects of mental disorder (3, 4); however, it also involves a process of personal change, as the person resumes their life project and recovers their maximum abilities and identity (4) as an individual and as a citizen (5). The interest in understanding the personal recovery process is growing, especially in English-speaking countries (6). One of the most widely accepted theoretical frameworks within the paradigm of personal recovery is CHIME: Connectedness, Hope and optimism about the future, Identity, Meaning in life and Empowerment (7).

The strategies in the recovery approach include peer-to-peer programs implemented in mental health services. The support of peers is often critical to recovery, since it encourages the sharing of experiences, emotions, and thoughts (8, 9). Given their experience of mental suffering, peer support workers (PSWs) can help others to deal with situations of disorientation and can apply their experience to favor recovery (10, 11). Among people with mental health problems, mutual support can be useful in detecting mental health problems at early stages, in coping with diagnosis, and in promoting social coexistence with mental suffering, both during hospitalization and especially in the recovery process (9, 12, 13). The information shared is often more credible and meaningful to the person than that provided by mental health professionals, because it is immediately relevant and comprehensible (14).

In general, peer support promotes a model of well-being that focuses on capabilities and recovery rather than one that centers on symptoms of the disease (8). People who have had similar experiences can provide genuine empathy and validation. In the context in which the peer-to-peer technique is developed, users with mental health issues from two institutions received training to enable them to develop a professional role in the mental health care teams. In this way, the role of the PSW is someone with direct experience of the mental health issues and meets a set of specific requirements to become part of the organization.

### Purpose

The fact of being able to adapt the training and materials of other experiences of international success will allow to launch a whole series of actions to improve the attention to mental health in our territory. Likewise, the fact of carrying out an adaptation of training activities validated in other countries could generate the start of a regulated training structure that would contribute to improving the quality of care for people with mental health problems. The project follows objective 2.4 of strategic line 2 of the Mental Health and Addictions Master Plan (MHAMPS). Strategies 2017–2020 of Catalonia (15) “Guarantee the participation of people with mental health problems and the organizations that represent them.” The study raises the following hypotheses:

1. The incorporation of people with mental health problems will improve community functioning and promote the quality and satisfaction of life of people with severe mental health problems.
2. The incorporation of people with mental health problems, trained, as PWS will have a positive impact on their own well-being as well as that of the affected people they support.
3. The incorporation of people with mental health problems will help to decrease the perception of social stigma toward the people affected.

## Materials and Methods

### Study Design

This study evaluated the impact of the implementation of the peer-to-peer technique using a mixed methods approach. The evaluation was carried out at three time points: pre-test (T1), post-training (T2), and post-test (T3).

The study protocol was assessed by an independent clinical research ethic committee. Reporting adhered to the guidelines for Journal Article Reporting Standards for Qualitative Primary, Qualitative Meta-analytic, and Mixed Methods Research in Psychology (16).

### Participants

The project included three groups of participants, with the following inclusion criteria:

1. PSWs: age 18–65, diagnosis of severe mental disorder, and optimal recovery process in the last year.
2. Service users: age 18–65, regular attendance at the Community Recovery Services (CRS), and diagnosis of severe mental disorder. The most frequent diagnoses were schizophrenia, bipolar disorder, and depression.
3. Professionals: age 18–65, CRS staff members, and with a range of profiles: psychologists, nurses, social workers, and occupational therapists.

Participants as PSWs (*n* = 16) were selected by their reference professionals, who contacted the candidates and explained the project to them. They were people in care follow-up and without any experience as PWSs. At all times, participants were informed of their rights, duties, and obligations: What does the treatment consist of? What is it for? How it is performed? What effects can it produce? What are its benefits? What risks does it have? The key element was their willingness to participate; it was made clear that participation was voluntary and that they could leave the program at any time. The climate was one of continuous dialogue, with the aim of finding a balance between personal needs and the proper functioning of the project. The PWS received financial compensation through a salary scholarship.

### Intervention

The project was carried out in central Catalonia, at two institutions providing mental health care—Osonament of Vic and the Division of Mental Health of the Althaia Foundation of Manresa. Osonament offers CRS: integrated community services specializing in the prevention and care of mental health and addictions, promoting comprehensive development, autonomy, and improvement of the person's quality of life. They accompany people to create a significant life project, favoring community integration; promote job placement through individualized job counseling; and offer home support so that the person can develop their life project in the most autonomous way. Althaia Foundation carries out a comprehensive social and health care plan for people with mental health problems and addictions. The community use of the spaces is facilitated, which favors integration and contributes to fighting against social stigma.

The main objective is to train, accredit, and integrate people with mental health problems in the mental health care teams. It is necessary to say that the participating professionals from each of the entities have held periodic coordination meetings throughout the development and implementation of the project, agreeing at all times on the actions to be carried out. In addition, the project has a collaboration with the first-person movement in mental health, ActivaMent Catalunya, which has a presence in the training block: a support in the preparation and writing of teaching materials and teaching tasks.

The process of adapting the peer-to-peer technique had three different phases: (1) theoretical training, (2) incorporation in the mental health teams, and (3) work as PSWs.

#### Theoretical Training

In order to adapt the peer-to-peer technique, different experiences of international success were sought, which could be suitable for the territory in which they were to be implemented. The theoretical contents of the training were elaborated from the previous selection of materials: (1) introduction (work environment, definitions, and objectives; (2) concept of recovery; (3) resilience; (4) ethics and rights; (5) communication skills; (6) mental health and addictions and social and health care network; (7) risks and limits of the intervention; (8) stigma, social participation, and citizenship; (9) troubleshooting; and (10) evaluation. Writing of the materials was carried out by the professionals of the participating entities (Osonament—Althaia Foundation—University of Vic-Universitat Central de Catalunya) and the first-person movement (ActivaMent). The contents of the theoretical training for the adaptation of the peer-to-peer technique were agreed upon by all the agents participating in their design and were configured as follows: 10 weekly sessions of 4-h duration. The sessions were held alternately in Manresa and Vic. It was attended by 16 students (equal percentage between Osonament and Fundación Althaia). To access work practices, 80% attendance was required as a requirement. It took place at the University of Vic-University of Central Catalonia, and all the PSWs received an accreditation by the university.

#### Incorporation to the Mental Health Teams

Once the theoretical training was completed, the people who met the specified criteria joined the mental health care teams as peer support agents of the two care provider entities: the Althaia Foundation's Mental Health Division and Osonament. Although most of the insertion occurred in CRS, insertion also occurred in an inpatient unit. We understand CRS as a free public rehabilitation community service that offers care to adults (between 18 and 65 years old) with serious mental disorders in which the personal, family, and social rehabilitation of the person is worked on. The CRS is that space located within the community, which allows the person to develop life projects in their environment.

#### Work as PSWs

People joined the care teams and worked over a 6-month period for an average of 10 h per week. PSWs joined a variety of services or intervention programs (case manager—therapeutic group management—hospitalization), and their participation was closely supervised by reference professionals at each site. In Table 1, we offer the list of tasks executed by the PWS. A weekly follow-up of 1 h is established between the reference professional and the PWSs.

**Table 1:** List of tasks executed by the PWS.

Review and preparation of documentation
Support for the Harm Reduction Program (aimed at people with substance use disorders)
Support for the Abstinence Support Program (aimed at people with substance use disorders)
Support for the Emotional Support and Activation Program
Support for the organization of Sports Days
Walking group
Relaxation workshop through meditation
Communications at conferences and congresses
Individual accompaniments
Supervisions with reference in the entity
Organizational meetings
Individual support through music of home residence
Home-residence music workshop
Support for the Functionality Support Program
Support for the Functional Rehabilitation Program
Support for the time occupation support program
Anti-stigma project design
Support for sports activities
Group Support Activities of Daily Living
Community accompaniment
Support for the home autonomy program
Support for the inpatient rehabilitation program
Individualized accompaniment to the community in hospitalizations
Support in the reception of the center
Support in the reception and dynamization of the social club
Support in the preparation and implementation of work dynamics
Participation in meetings of the Individualized Support Program

### Outcome Variables and Measures

Data on self-stigma, life satisfaction, participation in relevant activities, personal recovery, occupational performance, and attitudes toward mental health were recorded using the five questionnaires listed below:

The Self-Stigma Questionnaire (SSQ) (Cronbach's alpha ranging between α = 0.75 and ω = 0.901) uses the following Likert response categories: 1 = strongly agree, 2 = moderately agree, 3 = slightly agree, 4 = neither agree nor disagree, 5 = slightly disagree, 6 = moderately disagree, and 7 = strongly disagree. Higher scores indicate lower self-stigma (17).

The Spanish version of the Scale of Satisfaction with Life (SWLS) by Dieneret al., adapted by Atienza et al. (Cronbach's α = 0.88), offers an overall judgment of people's satisfaction with their own lives. It consists of five Likert-type items with scores ranging from 1 “strongly disagree” to 5 “strongly agree” (18, 19).

The Engagement in Meaningful Activities Survey (EMAS) (Cronbach's α = 0.91) reflects multiple proposals for occupational therapy and occupational science that address constituents of meaningful engagement. The EMAS addresses the assessment of the meaning of an occupation by bringing together diverse viewpoints on meaning and employment (20).

The Recovery Assessment Scale-revised (RAS-R) (Cronbach's alpha ranging between α = 0.93 and ω = 0.95) is a self-applied instrument that measures personal recovery, developed over 20 years ago by Gifford and colleagues in the United States. The RAS-R consists of 24 items on a five-level scale “strongly disagree,” “disagree,” “not sure,” “agree,” and “strongly agree” (21).

The Canadian Occupational Performance Measure (COPM) is an individual assessment tool designed to detect changes in clients' self-perceptions of their performance and satisfaction over time. It is scored with values from 1 (lowest) to 10 (highest score) (22). Test–retest reliability of the COPM was *r* = 0.842.

The Community Attitudes toward Mental Illness (CAMI) by Taylor and Dear is a scale composed of 40 items, rated on a 5-point Likert scale, ranging from total agreement to total disagreement (Cronbach's alpha ranging between α = 0.861 and ω = 0.909). The scale consists of four named factors: authoritarianism, benevolence, social restraint, and community health mental ideology, each of which contains 10 statements regarding opinions on how to treat and care for people with severe mental illness. Five of these 10 items are expressed positively and the other five negatively (23).

These methods were chosen considering two criteria: (1) they measure results in accordance with the personal recovery paradigm and (2) instruments were validated into Spanish.

### Data Collection Procedures

At the beginning of the training (T1), sociodemographic data were collected from the PSWs, and outcome measures were administered to all participant groups. After 6 months (T2), the PSWs who had successfully completed the training answered the same questionnaires and additionally the COPM. Finally, at 12 months (T3), all participants were contacted again to complete the outcome measures and the COPM. At the end of the program, focus groups were used to further assess the impact on the participants. All the professionals at the two reference institutions where PSWs were included were contacted *via* an online questionnaire with an open-ended item format (24). At the same time, focus groups (25) were held with the PSWs and service users, based on a format of semi-structured, open-ended interviews and lasting an average of 90–120 min. The questions revolved around perceptions about the program and their own execution of the PSWs' role, the perceived impact on their own recovery process, and suggestions for the implementation of future programs.

Questionnaire and focus groups were carried out in Catalan and Spanish, and literal transcriptions were produced for their final analysis. Only the quotations selected for this manuscript have been translated into English (Table 2 shows the assessment levels and the data collection measures).

**Table 2:** Outline of the mechanisms used for analysis.

	**Time 1**			**Time 2**			**Time 3**		
	**PWS**	**SU**	**MHP**	**PWS**	**SU**	**MHP**	**PWS**	**SU**	**MHP**
SSQ	X	X		X			X	X	
SWLS	X	X		X			X	X	
EMAS	X	X		X			X	X	
RASR	X	X		X			X	X	
COPM	X						X		
CAMI			X						X
FG							X	X	
Q									X

*PSW, peer supporter worker; SU, service user; MHP, mental health professional; SSQ, Self-Stigma Questionnaire; SWLS, Satisfactions With Life Scale; EMAS, Engagement in Meaningful Activities Survey; RAS-R, Recovery Assessment Scale-revised; COPM, Canadian Occupational Performance Measure; CAMI, Community Attitudes toward Mental Illness; FG, focus group; Q, questionnaire*.

### Data Analysis

#### Quantitative Measures

Two researchers (PV and CP) used the SPSS software (version 28.0). A repeated measures analysis of ANOVA was used on the instruments with three data collection points. Using Cohen's *d*, the effect size of the mean differences was verified. The analysis of the data was completed using non-parametric tests, the Wilcoxon rank test for repeated measurements and the Mann–Whitney *U* test for mean comparisons between groups. In all cases, a *p*-value < 0.05 was considered to reject the null hypothesis.

#### Qualitative Interviews

Three independent researchers (SA, PV, and CP) used the Atlas.ti (version 9.1) to make the analysis of the narratives and to create category groups, which were analyzed. The content analysis technique was used. This began with an exhaustive reading of the transcripts of the first interviews with each participant, carried out by the first author. After this reading, the material was coded, and the quotations were grouped in relation to their similarity and in relation to the objective of the study, through several discussions between the researchers. Based on these discussions, preliminary groups of codes were generated, which were compared by the researchers until reaching more central themes. Ten PSWs, who completed the project from start to finish, participated in the focus groups. Twelve professionals participated in the online questionnaires evaluating their participation in the program. Twenty-one service users participated in the focus groups.

#### Data Integration

The mixed method used was the convergent parallel design (26). The triangulation of the qualitative and quantitative findings facilitated the understanding of the results and showed its coherence and lack of contradictions. It is a methodology that allows the convergence of different data: beneficial to provide confirmation of findings, more complete data, greater validity, and better understanding of the studied phenomena (27). A methodological triangulation was carried out. When using different methods in triangulation, the aim is to analyze the same phenomenon through different approaches.

## Results

**Figure 1:**
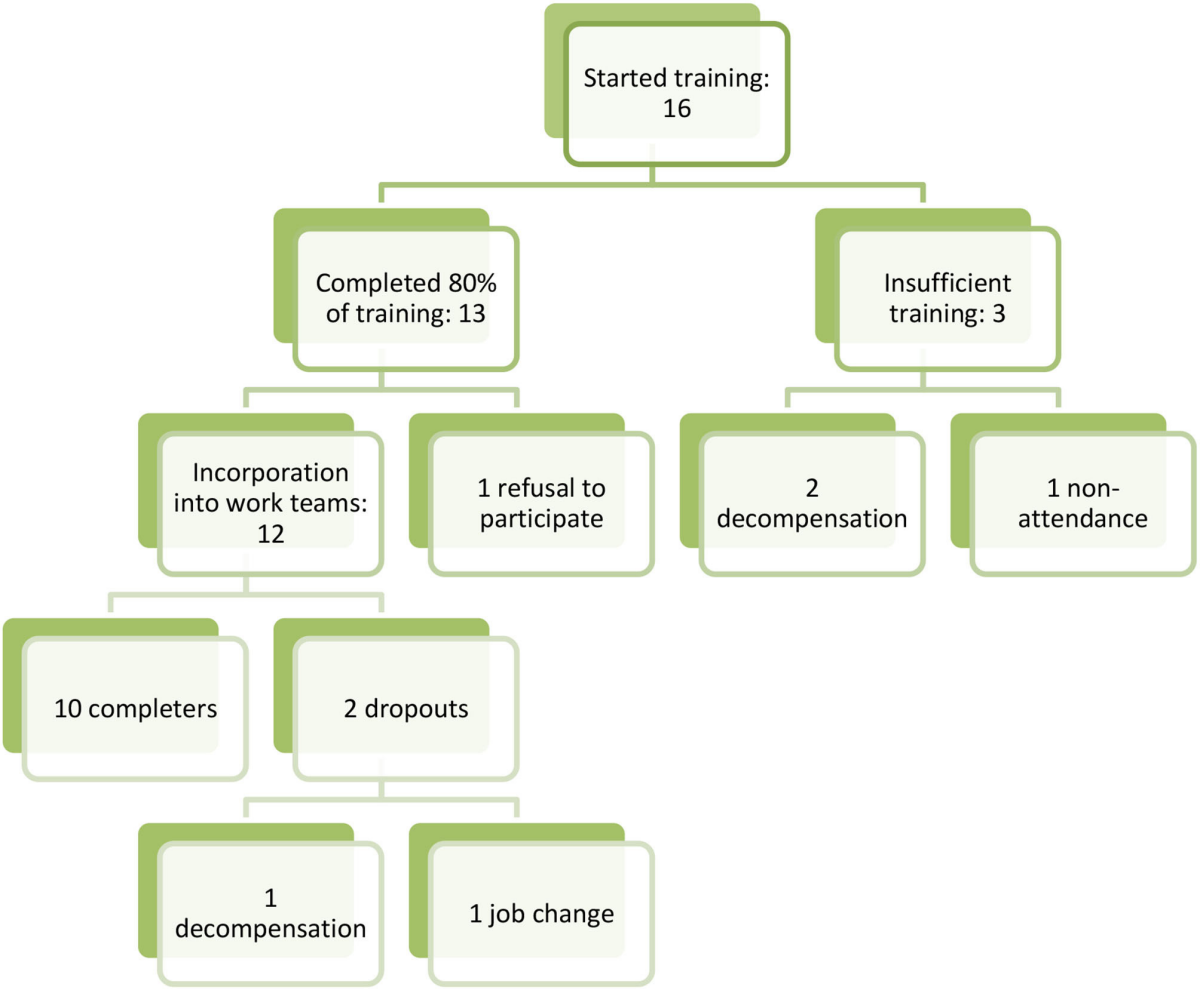
Peer-to-peer insertion process.

Initially, there were 16 PSWs, divided equally between the two institutions. Ten PSWs eventually completed the whole project, from the training phase to the placement in the work teams (see Figure 1).

These 10 people were the only ones who completed the entire project.

### Participants and Intervention

The study population comprised 162 participants (professionals, *n* = 69 (47%); PSW, *n* = 16 (19%); service users, *n* = 77 (43%); Table 3). Women accounted for 52% of the sample. Most participants were aged between 36 and 55, and 5% were under 25.

**Table 3:** Baseline demographics.

		**PSW**	**SU**	**MHP**
		**16**	**77**	**69**
Female		9 (56%)	52 (68%)	52 (75%)
**Age**				
	<25	–	/	6 (6%)
	26–35	–	/	20 (20%)
	36–45	2 (12%)	/	14 (39%)
	46–55	7 (44%)	/	14 (20%)
	>56	7 (44%)	/	15 (15%)

*PSW, peer supporter worker; SU, service user; MHP, mental health professional*.

### Qualitative Results

There were 583 quotations explored, which were grouped into 68 codes, and these were under the umbrella of four groups of codes. Four groups of categories have been created considering the content of the story, i.e., the first level of categorization has been created under the premise “Who or what are you talking about?” Therefore, the first group refers to the task of ACCOMPANY (A), followed by PEERS (P), all those stories that focus on the practice of PSWs; below, we find the group MENTAL HEALTH PROFESSIONALS (MHP), all those stories that focus on the professionals of the teams); and finally, the PROJECT group (PRO), all those stories that focus on the phases of the project (proposals for improvement have been collected here). In all the categorical groups, we find stories of all the participants (see Table 4).

**Table 4:** Qualitative analysis: codes, group of codes, and quotations.

**Code group**	**Codes**	**Quotations**
Accompany		212
	*Stigma*	24
	*Understanding empathy*	23
	*Hope*	18
	*Support*	14
	*Values*	14
	*Complement*	13
	*Recovery*	8
	*Opening*	7
	*Confidence*	7
	*Misunderstanding*	7
	*Motivation*	7
	*Proximity*	7
	*Learning*	5
	*Listen*	5
	*Equal accompaniment*	4
	*Self-stigma*	4
	*Self esteem*	4
	*Identification*	4
	*Inclusion*	4
	*Relationship*	4
	*Acceptance*	3
	*Social impact*	3
	*Life quality*	3
	*Taboo*	3
	*Training*	2
	*Tip*	2
	*Previous experience*	2
	*Individuality*	2
	*Investment*	2
	*Normalization*	2
	*Suffering*	2
	*Inspiration*	1
	*Resources*	1
	*Resilience*	1
PEERS		206
	*Impact by peer*	34
	*Functions*	31
	*Example*	30
	*Lived experience*	27
	*Empowerment*	17
	*Feeling useful*	13
	*Meaning*	12
	*Meaningful employment*	10
	*Fortress*	6
	*Nervous*	5
	*Employment*	5
	*Be like professionals*	5
	*Substitution*	3
	*Wellness*	2
	*Stress*	2
	*Previous experience*	2
	*Self-realization*	1
	*Satisfaction*	1
Mental health professionals		22
	*Impact for the professional*	8
	*Concerns*	5
	*Reality theory discrepancy*	4
	*Medication*	3
	*Break down barriers*	2
Project		143
	*Improvements*	30
	*Positive assessment*	28
	*Challenge*	23
	*Concern*	18
	*Training*	16
	*Innovation*	14
	*Ambiguity*	6
	*Task planning*	4
	*Uncertainty*	2
	*Information*	1
	*Remuneration*	1

Group A (212 quotations) is made up of 34 codes. It is observed that the categories Stigma and Comprehension–Empathy are the ones that include more stories, 24 and 23 quotations, respectively. Below, we find the Hope category with 18. Next, we find how Support and Values, with 14 each, and Complement, with 13, are the most references. The remaining 23 codes are in a range between eight and one quotation.

Group P (206 quotations) is made up of 18 codes. There are three categories above the rest: Impact by peer (28), Functions (29), and Example (30). In a step below is the code Lived Experience and Empowerment, with 27 and 17 quotations, respectively. The rest is in a fork between 12 (Meaning) and one story (Self-realization).

The MHP group (22 quotations) is made up of five codes. It has been a little referenced group, and we would highlight Impact for the professional with eight quotations. It is followed by Concerns, Reality Theory Discrepancy, Medication, and Breaking Barriers in order of quotations.

The PRO group (143 quotations) is made up of 11 codes. The category Improvements has been the most referenced with 30 quotations, followed by Positive evaluation with 28 quotations, and Challenge with 23. Behind, we would find a second group between the fork of 16 and one quotation in which the categories Training and Innovation with 16 and 14 quotations, respectively, stand out.

### Contribution of the Project to the Personal Recovery Process

Data on the project's impact come from the quantitative measures and qualitative interviews (see Tables 5, 6).

**Table 5:** Quotations used for mixed analysis.

	**Label**	**Narrative**
PWS	Perception of life satisfaction	“*[...] it made me feel very good. In fact, at the weekend I missed it [...]*
		*[...] it's an important help in our professional goal of improving [...]*
	Nerves and stress	*[...] At the beginning I felt a bit nervous […]*
		*[...] I felt a bit of pressure but for myself [...]*
		*Right now I want to finish because it is making me feel a little [...]*
		*Yes, there are days when I get stressed [...]*
	Ability to find work, work as a PSW, and giving to the community	*at the same time you discover skills that you didn't know you had [...]”*
		*I came out strengthened and it gave me a lot of confidence [...]*
		*but it has opened up my mind and showed that I can work on something [...]*
		*It is a very good way to make the peer feel motivated, and empowered [...]*
		*It's made me feel more confident of myself / and not see myself as” sick “[...]*
	Employment	“*[...] Sometimes a job is so hard you just say “I'm not interested [...]*
		*[...] having an occupation, not just a job... very often [...]*
		*[...] I've been in jobs where I was very busy but what I was doing was pointless*
		*[...] With peer2peer I feel that I've got a direction [...]*
		*[...] When I come to Mosaic I enjoy myself, it's very rewarding [...]*
	Meaningful occupation	*[...] having an occupation, not just a job... very often [...]*
		*[...] I've been in jobs where I was very busy but what I was doing was pointless [...]*
		*[...] With peer2peer I feel that I've got a direction [...]*
		*[...] When I come to Mosaic I enjoy myself, it's very rewarding [...]*
	Sense of being useful	*[...] I use my most personal skills for the good of the group. And this [...]*
		*[...] all this is what gives it meaning. These moments when you see that it is [...]*
		*[...] participating in peer2peer comes to my mind that I can make a contribution [...]*
		*[...] It sounds obvious... but we felt useful [...]*
		*[...] I think it provides a sense of usefulness and involvement [...]*
	Self-esteem	*[...] Very often doing an occupation that suits you creates self-esteem […]*
		*[…] when you find a reason for doing something then you can do it [...]*.
SU	Hope and optimism	*First of all optimism, because you see that they're dealing very well with the situation, they're happy […]*
		*I know that you can have a disorder but still be happy*.
	Understanding and empathy	*[…] when you go to the professional, you think “I don't know if he will understand me” […]*
		*And there's an empathy that is difficult to find with other people*.
		*I believe that people like us feel more trust for a person who has gone through the same thing […]*
		*You have to go through it (share it) if you don't, you don't see anything in your heart*.
		*[..] it is easier to talk about these things with PSWs […]*
		*Being with a person with a mental difficulty like you makes you feel safer*
	Inspiration and example	*I feel positive...seeing that you can suffer from a disorder but still be ok afterwards […]*
		*He accepted the challenges and overcame them. And I just want to do the same […]*
		*He comes from the real world is and is positive, he can motivate people […]*
		*I also think that being able to be a support agent helps. [...]*
		*Because you are helping other people and that for me multiplies the recovery [...]*
		*Feeling useful is essential in life*.
		*[...] It must help a lot to feel useful and alson [...]*
MHP	Acceptance	“*I didn't regard the PSW like an intern or a user of the program, just another team member providing support on a day-to-day basis*”
		“*Personally it has been enriching, I have learned from seeing other views of the same problem and the opportunity for change from a particular* person”
		“*It has been very positive, I have felt very comfortable at all times. A member of the team*”
	Personal and professional growth	“*A synergistic learning process*”
		“*I think it is very positive for professionals to see that there are people with a disorder who can later help others on their journey; they provide a vision and a way of working based on details and proximity, constancy*.”
		“*It greatly enriches professional work; they help us to provide better service and are a source of satisfaction for the majority of users*”
	Power of change	“*Being able to break the barriers of professional–patient* [...]”
		“[...] *working on stigma, admitting the shortcomings of the system, not overvaluing professional decisions, respecting the decisions of the patients and, above all, recognizing that society is diverse*”
		“[...] *accept that mental health problems are another characteristic of the people with whom we share spaces. Work, life, etc*. [...]”

**Table 6:** Results grouped according to levels of participation.

**Assessment**		**Time 1, mean**	**Time 2, mean**	**Time 3, mean**	***p*-value**	**Effect size**
		**(SD)**	**(SD)**	**(SD)**		***d* (Cohen's) T1–T3**
**PWS**						
	SSQ	80.60 (12.59)	78.50 (11.64)	80.11 (16.49)	0.294	−0.03
	SWLS	23.73 (5.70)	26.57 (6.07)	22.22 (4.99)	0.748	−0.30
	EMAS	48.53 (7.52)	53.25 (4.36)	50.88 (6.41)	0.162	0.37
	RASR	96.42 (8.98)	101.50 (9.31)	97.33 (7.46)	0.093	0.12
	Occupational development: ability to find work	6.92 (1.97)		7.08 (2.50)	0.038	1.00
	Occupational development: working as a support agent	7.19 (1.68)		8.38 (1.19)	0.016	1.08
	Occupational development: giving to the community	7.50 (1.63)		8.54 (0.96)	0.011	0.06
	Satisfaction: ability to find work	7.43 (2.10)		8.42 (1.44)	0.031	0.69
	Satisfaction: working as a support agent	8.56 (1.89)		9.23 (1.01)	0.180	0.66
	Satisfaction: giving to the community	8.75 (1.57)		9.31 (0.75)	0.063	0.75
**SU**						
	SSQ	57.15 (18.03)		59.48 (17.89)	0.702	0.13
	SWLS	16.71 (7.35)		18.18 (7.09)	0.954	0.21
	EMAS	41.33 (10.07)		44.13 (10.49)	0.810	0.27
	RASR	80.47 (12.09)		79.70 (17.97)	0.648	−0.04
**MHP**						
	*CAMI*	21.62 (2.02)		21.67 (2.55)	0.676	0.02
	*Authoritarianism* pro					
	*CAMI*	9.74 (2,58)		9.61 (2,32)	0.343	−0.38
	*Authoritarianism* anti					
	*CAMI*	8.45 (2.12)		8.76 (2.48)	0.750	0.13
	*Benevolence* pro					
	*CAMI*	22.46 (1.55)		22.05 (2.23)	0.277	−0.18
	*Benevolence* anti					
	*CAMI*	23.04 (1.90)		22.28 (2.16)	0.124	−0.35
	*Social restrictiveness* pro					
	*CAMI*	8.82 (2.23)		9.30 (2.72)	0.648	−0.15
	*Social restrictiveness* anti					
	*CAMI*	10.10 (1.80)		10.56 (1.99)	0.247	0.23
	*Community mental health ideology* pro					
	*CAMI*	22.57 (1.97)		22.81 (2.41)	0.435	0.10
	*Community mental health ideology* anti					
	*CAMI* Total	124.25 (4.94)		124.71 (5.50)	0.628	0.08

*PSW, peer supporter worker; SU, service user; MHP, mental health professional; SSQ, Self-Stigma Questionnaire; SWLS, Satisfactions With Life Scale; EMAS, Engagement in Meaningful Activities Survey; RAS-R, Recovery Assessment Scale-revised; COPM, Canadian Occupational Performance Measure; CAMI, Community Attitudes toward Mental Illness; p < 0.05*.

### PSWs

#### T1–T2

The training had a positive impact on the participants.

##### Self-Stigma

The results of the questionnaire showed a decrease of 2.1 (T1 = 80.60–T2 = 78.50) in the perception of perceived stigma (*p* = 0.344). In turn, the changes were detailed on the Satisfaction with Life Scale (*p* = 0.586) with an increase of 2.84 points between the two periods (T1 = 23.73–T2 = 26.57). Regarding the survey on participation in significant activities, an increase of 4.72 (T1 = 48.53–T2 = 53.25) was observed at the end of training (*p* = 0.753). The greatest differences observed occurred in the revised Recovery Assessment Scale (T1 = 96.2–T2 = 101.50) with an increase of 5.3 in the perception of personal recovery (*p* = 0.446). However, the collected changes did not show statistically valuable differences (*p* < 0.05).

#### T2–T3

The data collected at the end of the project show us a decrease in all the measures used in the three times with the exception of SSQ. A statistically significant decrease (*p* = 0.042) was observed in the Satisfaction with Life Scale with a reduction of 4.35 (T3 = 22.22). An increase in the perception of stigma (*p* = 0.893) of 1.61 (T3 = 80.11) is observed. With respect to EMAS (*p* = 0.750) and RASR (*p* = 0.528), decreases of 2.37 (T3 = 50.88) and 4.17 (T3 = 97.33) were registered, respectively.

The effect of the commented differences has a small effect. All of them are less than *d* = 0.50.

#### Canadian Measure of Occupational Performance

Three performance parameters were analyzed in which significant improvements were obtained: ability to find work (*p* = 0.038), work as a support agent (*p* = 0.016), and give to the community (*p* = 0.011). Regarding satisfaction, three parameters were registered, obtaining significant improvements in the ability to find work (*p* = 0.031). There were improvements in the remaining parameters but without significant values: working as a support agent (*p* = 0.018) and give to the community (*p* = 0.063).

With the exception of the perception in the performance of give to the community (*d* = 0.06), the subtraction of items presents an effect above *d* = 0.50, so we can affirm a medium effect in the differences.

- *PSW: “[...] it made me feel very good. In fact, at the weekend I missed it [...]*
- *PSW: [...] it's an important help in our professional goal of improving [...]*.

Two categories that might explain this decrease in the subjective perception of life satisfaction were nerves and stress. This decrease in the perception of life satisfaction can be observed in the results obtained in the qualitative analysis:

- *PSW: [...] At the beginning I felt a bit nervous […]*
- *PSW: [...] I felt a bit of pressure but for myself [...]*
- *PSW: [...] Right now I want to finish because it is making me feel a little [...]*
- *PSW: [...] Yes, there are days when I get stressed [...]*.

The perception of occupational performance improved in all the roles analyzed: ability to find work, work as a PSW, and giving to the community, which can relate directly to job placement. The PSWs experienced a sense of empowerment:

- *PSW: [...] at the same time you discover skills that you didn't know you had [...]”*
- *PSW: [...] I came out strengthened and it gave me a lot of confidence [...]*
- *PSW: [...] but it has opened up my mind and showed that I can work on something [...]*
- *PSW: [...] It is a very good way to make the peer feel motivated, and empowered [...]*
- *PSW: [...] It's made me feel more confident of myself / and not see myself as” sick “[...]*.

Also, in the field of occupational performance, PSWs presented an increase in satisfaction in the ability to find work, relating to their own practice as a peer agent. They saw it as a truly meaningful occupation. The qualitative results reveal three categories that exemplify this perception of fulfillment:

- *Employment: “[...] Sometimes a job is so hard you just say “I'm not interested [...]”*
- *A meaningful occupation: “[...] having an occupation, not just a job... very often [...] …… / “[...] I've been in jobs where I was very busy but what I was doing was pointless [...]” / “[...] With peer2peer I feel that I've got a direction [...]”/ “[...] When I come to Mosaic I enjoy myself, it's very rewarding [...]”*
- *Sense of being useful: “[...] I use my most personal skills for the good of the group. And this [...]”/ “[...] all this is what gives it meaning. These moments when you see that it is [...] / “[...] participating in peer2peer comes to my mind that I can make a contribution [...] / “[...] It sounds obvious... but we felt useful [...] / “[...] I think it provides a sense of usefulness and involvement [...]*.

Peer-to-peer practice was particularly meaningful for participants, and they themselves related it directly to an increase in self-esteem:

- *PSW: [...] Very often doing an occupation that suits you creates self-esteem […]*
- *PSW: […] when you find a reason for doing something then you can do it [...]*.

### Service Users

Improvements were obtained in all the measures used with the exception of the RAS. In the Self-Stigma Questionnaire, the improvement was 2.33 points higher than the baseline level (*p* = 0.702); the instrument that measures satisfaction with life (SWLS) registered a change of 1.47 (*p* = 0.954); and the EMAS detected an improvement of 2.8 (*p* = 0.810) compared to the start. The personal recovery variable suffered a setback compared to the initial one of 0.77 (*p* = 0.648), giving practically the same results. However, despite the aforementioned changes, none were of statistical value. The analysis shows a small-difference effect (*d* = 0.20).

Service users stated that the presence of PSW had been a source of hope and optimism:

- *SU: First of all optimism, because you see that they're dealing very well with the situation, they're happy […]*
- *SU: […] I know that you can have a disorder but still be happy*.

Service users also reported feeling closer to the PSWs and opened up more quickly. They expressed a greater degree of understanding:

- *SU: […] when you go to the professional, you think “I don't know if he will understand me” […]*
- *SU: And there's an empathy that is difficult to find with other people*
- *SU: I believe that people like us feel more trust for a person who has gone through the same thing […]*
- *SU: You have to go through it (share it) if you don't you don't see anything in your heart*
- *SU: [..] it is easier to talk about these things with PSWs […]*
- *SU: Being with a person with a mental difficulty like you makes you feel safer*.

PSWs were a source of inspiration and an example:

- *SU: I feel positive...seeing that you can suffer from a disorder but still be ok afterwards […]*
- *SU: He accepted the challenges and overcame them. And I just want to do the same […]*
- *SU: He comes from the real world is and is positive, he can motivate people […]*.

And, a model for recovery:

- *SU: I also think that being able to be a support agent helps. [...]*
- *SU: Because you are helping other people and that for me multiplies the recovery [...]*
- *SU: Feeling useful is essential in life*
- *SU: [...] It must help a lot to feel useful and alson [...]*.

### Mental Health Professionals

The results between the two-time units show stability over time. Globally, there has been an increase in the Community Attitudes toward Mental Illness scale of 0.46 (T1 = 124.25–T2 = 124.71) without statistical value (*p* = 0.628). The changes in the four factors (authoritarianism, benevolence, social restraint, and community health mental ideology) and the differences are below one point and without statistical value (*p* < 0.05). The analysis shows a small difference effect (*d* = 0.20).

The qualitative evaluation indicated that professionals fully accepted the PSWs and regarded them as colleagues:

- *MHP: “I didn't regard the PSW like an intern or a user of the program, just another team member providing support on a day-to-day basis*”
- *MHP: “Personally it has been enriching, I have learned from seeing other views of the same problem and the opportunity for change from a particular* person”
- *MHP: “It has been very positive, I have felt very comfortable at all times. A member of the team*”.

They also stated that the process of sharing workspace with a PSW has been a journey of personal and professional growth:

- *MHP: “A synergistic learning process*”
- *MHP: “I think it is very positive for professionals to see that there are people with a disorder who can later help others on their journey; they provide a vision and a way of working based on details and proximity, constancy*.”
- *MHP: “It greatly enriches professional work; they help us to provide better service and are a source of satisfaction for the majority of users*”.

Finally, they were well aware of the stigma suffered by people with mental health problems, and believed in the potential of PSWs to break down barriers:

- *MHP: “Being able to break the barriers of professional–patient* [...]”
- *MHP:* “[...] *working on stigma, admitting the shortcomings of the system, not overvaluing professional decisions, respecting the decisions of the patients and, above all, recognizing that society is diverse*”
- *MHP:* “[...] *accept that mental health problems are another characteristic of the people with whom we share spaces. Work, life, etc*. [...]”.

## Discussion

This pilot study explored the influence that peer interventions at mental health institutions had with the PSWs, the clients, and the professionals. On the one hand, it analyzed the effect of the program on PSWs and service users, quantifying the impact on life satisfaction, social participation, self-stigma, and occupational development. On the other hand, data from the professionals were collected about their attitudes toward people with mental health problems. The use of qualitative techniques facilitated a greater understanding of the impact of the program. This triangulation of results enabled the research questions to be addressed from different perspectives, thus providing a fuller picture than could be gained using a single method (31). Triangulation refers to the use of multiple methods or data sources in qualitative research to develop a comprehensive understanding of phenomena (30). It is a methodology that seeks to ensure that the results are consistent and not contradictory (29).

### PSWs

PSWs presented changes in the perception of satisfaction with life (SWLS), the development of occupational roles (COPM), and satisfaction in the ability to find work (COPM), between the start and the end of the study. The SWLS results reflect a reduction in the perception of satisfaction with life that we can attribute to the challenge of new roles and the obligation to modify a well-established routine. As Ibrahim et al. (32) noted in their systematic review, the failure to implement measures that promote the well-being of PSWs can be a barrier for future programs. Relapse in PSWs is one of the main concerns of mental health providers (33) and may be an obstacle to the implementation of programs of this kind. Indeed, one PSW dropped out due to a relapse in the recovery process. This same situation was observed in a pilot study in Scotland (28) in which 11 PSWs were rehospitalized. These findings suggest the need to develop strategies for promoting a healthy working role and ensuring a positive environment.

With reference to occupational performance, there were improvements in all the roles analyzed: ability to find work, work as a support agent, and giving to the community, which can be related directly to the job placement. This situation was already noted by Hutchinson et al. (34) and may reflect the PSW's perception of becoming a socially valuable citizen. In the focus groups, PSWs noted the enormous benefit the practice has brought to their lives: it gave them a reason to continue the process of personal recovery and a sense of usefulness. They were keen to work as PSWs in the future and considered it to be an attractive and meaningful job option. Similarly, in their interviews with PSWs, Salzer (35) recorded that the participants felt a greater sense of self-esteem and personal growth due to the opportunity to do something useful. Simó and Guzman (36) emphasized the importance of an intervention through the construction of life projects and the possibility to engage in meaningful occupations. Many people do not know it, but everyone wants to be excited about something. Significant occupations have an impact on self-concept of self-efficacy (37, 38).

### Service Users

Service users attended by PWSs did not show significant changes in the different measures of life satisfaction, meaningful activities, and level of recovery or social self-stigma. However, in the discussion groups, they described the presence of the new figure in the teams as very positive. They noted that it helped them to resolve doubts, increased their motivation, and encouraged them to express their suffering and to develop a more positive attitude. They felt more empowered and increased their social relationships. Other studies that have explored the figure of PSW recorded significant changes. Corrigan et al. (39) adapted an agent support program taking into account the cultural origin of the population served and obtained significant results in empowerment. Similarly, in a program deployed in Germany, Mahlke et al. (40) observed significant improvements in the quality of life, and Cook et al. (41) obtained significant changes in personal recovery and hope. Our project observed improvements in three of the explored areas (SSQ–SWLS–EMAS) and a slight decrease in RAS. However, the effect of the differences is very low. This makes us think about the design of future projects, considering the time linked to the service and comparing the data with a larger sample.

### Professionals

The quantitative data did not indicate changes in the level of stigma toward mental health; in fact, the values obtained showed the level of stigma to be very low compared to other population groups (42). The professionals at the two institutions have extensive experience, which is also likely to keep stigma at a low level. Professionals highlighted the fluidity in the incorporation of PSWs into the teams and the acceptance of their new role in mental health care. This situation draws attention to a key element in the program's implementation, namely, the professionals' acceptance of the new figure (32). In addition, there is a professional enrichment that translates into an improvement in praxis. PWSs in mental health is one of the central strategies within the recovery model (43). Their insertion in work teams has a catalytic role in the implementation of the new paradigm (10). PWSs can detect stigmatizing attitudes on the part of professionals. The study of Mancini (44) shows how, despite the fact that PWSs were members of the staff, they suffered stigmatizing behavior from the MHPs. Interventions must empower PWSs with autonomy and power in order to raise their voices and transform the system (45).

## Limitations

The small sample size may have limited the scale of the changes obtained. Furthermore, the time elapsed between the initial and final evaluations was not the same in all participants; the resources are dynamic, and the patients come and go at different stages in their process and, in fact, are seen at different levels of care (some in the hospital setting, others during the recovery phase in the community). This means that not everyone is in the same recovery process and social situation.

## Recommendations for Practice

The incorporation of people with mental health experience is one of the objectives of the MHAMPS. This project has been an innovative experience in the incorporation of PWS mental health teams in Catalonia. The study's findings suggest that one of the key factors is support in employment. The results have shown us a decline in the perception of quality of life. It will be very important in future projects to be able to detect this burnout and accompany the PWS in building strengthening strategies.

The combination of quantitative instruments with qualitative data collection strategies allows a greater understanding of reality. At the same time, the creation of spaces for exchange serves participants to reflect on the impact on their lives.

The project lays the groundwork for an RCT at the territorial level, taking advantage of the design of the study and the outcome measures. This would make it possible to generate stronger territorial evidence and greater pressure on political authorities to implement these programs.

## Conclusion

The present study has involved a whole series of reflections and considerations that may have a bearing on the development of peer-to-peer programs, offering new employment opportunities for people with mental health. Despite the difficulties that have arisen in the implementation of this pilot project, the experience was satisfactory for all participants, and the impact on the PSWs and on service users was highly meaningful.

Peer-to-peer project opens up an employment opportunity for people with mental health problems and appears as a meaningful occupation for them. The practice of PSWs can play a key role in the recovery process: it offers a feeling of usefulness for the PSWs and brings hope to the clients showing that recovery is possible. This strategy collaborates in the implementation of the recovery model in mental health institutions. Further analyses are now needed to study ways of encouraging the incorporation of PSWs in mental health care.

This is an example of the importance of accompanying recovery processes through the construction of life projects.

## Data Availability Statement

The raw data supporting the conclusions of this article will be made available by the authors, without undue reservation.

## Ethics Statement

The study protocol was assessed by an Independent Clinical Research Ethics Committee. The patients/participants provided their written informed consent to participate in this study. Written informed consent was obtained from the individual(s) for the publication of any potentially identifiable images or data included in this article.

## Author Contributions

All authors listed have made a substantial, direct, and intellectual contribution to the work and approved it for publication.

## Funding

This work was supported by the [Catalan Agency for Management of University and Research Grants] under Grant [2019-DI-012] and [La Caixa Fundation] under Grant [IS18-00323].

## Conflict of Interest

The authors declare that the research was conducted in the absence of any commercial or financial relationships that could be construed as a potential conflict of interest.

## Publisher's Note

All claims expressed in this article are solely those of the authors and do not necessarily represent those of their affiliated organizations, or those of the publisher, the editors and the reviewers. Any product that may be evaluated in this article, or claim that may be made by its manufacturer, is not guaranteed or endorsed by the publisher.
