# Supplementary material for: Training Peer Support Workers in Mental Health Care: A Mixed Methods Study in Central Catalonia
Source: Front Psychiatry. 2022 Apr 8;13:791724. doi: 10.3389/fpsyt.2022.791724 (PMC9023875; doi:10.3389/fpsyt.2022.791724)
Supplement: Supplementary file 1 [file Data_Sheet_1.pdf]

Annex 1 - FOCUS GROUP script

1. We start the focus group by asking the participants to introduce themselves and explain what they think the program is appropriate for them. The moderator will also introduce himself. We want to generate a cordial, relaxed atmosphere.

2. We explore participants' expectations of the support worker program, their familiarity with programs of this type, their hopes or suspicions about the technique, etc. This question serves as a starting point for our later reflections on the evolution of the program.

3. Next, we aim to capture the impact of the training process, and whether it helped their development as PSWs.

4. Next, we focus on their practice as PSWs at the service to which they were assigned, their first impressions, the added value they provided, etc.

5. We assess the impact of their participation as PSWs on their life process (i.e., recovery); i.e., if they were able to bring to the patient something that they themselves had not received at the time of their illness.

6. Finally, we ask the group about the viability of the program: future challenges, why the program is important, etc.

Annex 2 - Script Professional questionnaire

1. Initial expectations. Every innovative project takes us out of our comfort zone.

a. What were your first impressions when the project was described to you?

b. Is there anything that worries you? If so, please specify.

2. Description of the service. The peer-to-peer intervention strategy aims to take advantage of first-hand experience and the integration into work teams after personalized training.

a. What are the challenges you have encountered in your day-to-day life?

b. Which elements have been the most positive?

c. Which elements do you think can be improved?

d. What added value did the PSWs brought to the rehabilitation process of the people cared for?

e. What roles did they play?

f. Do you think they can play other roles in the service? If so. specify which.

3. Personal impact. One of the features of peer-to-peer is its reciprocal impact between the person receiving the service, the PSW and the professional.

a. What do you think this professional experience has brought to you on a personal level?

b. Has the opportunity to participate in the program impacted your psychological well-being? How?

c. Has it been positive for you to share workspaces with peers and accompany processes of autonomy with people diagnosed with mental health problems?

4. Challenges for the future. As we have mentioned, the proposed strategy is an innovative tool that is not widely used at present.

a. Why do you think the presence of peers on mental health teams is important?

b. Do you think their presence should be increased? Why?

c. Do you think it helps to break down the stigma related to mental health? If so, why?

d. What challenges do we have (as a society) with regard to spreading the use of this practice?
